# Collagen V oral administration decreases inflammation and remodeling of synovial membrane in experimental arthritis

**DOI:** 10.1371/journal.pone.0201106

**Published:** 2018-07-30

**Authors:** Silvana Ramos Atayde, Ana Paula Pereira Velosa, Sergio Catanozi, Vanessa Del Bianco, Priscila Cristina Andrade, José Eduardo de Castro M. Rodrigues, Antonio dos Santos Filho, Leila Antonangelo, Suzana Beatriz Veríssimo de Mello, Vera Luiza Capelozzi, Walcy Rosolia Teodoro

**Affiliations:** 1 Department of Pathology, Faculdade de Medicina FMUSP, Universidade de Sao Paulo, Sao Paulo, São Paulo, Brazil; 2 Rheumatology Division, Faculdade de Medicina, Universidade de Sao Paulo, Sao Paulo, São Paulo, Brazil; 3 Endocrinology Division (LIM 10), Faculdade de Medicina FMUSP, Universidade de Sao Paulo, Sao Paulo, São Paulo, Brazil; University of South Florida St Petersburg, UNITED STATES

## Abstract

Because collagen type V (Col V) can be exposed in tissue injury, we hypothesized that oral administration of this collagen species modulates the inflammation and remodeling of experimental synovitis, avoiding joint destruction, and that the modulation may differ according to the temporal administration. Arthritis (IA, n = 20) was induced in *Lewis* rats by intraarticular (ia) injection of 500 μg of methylated bovine serum albumin (mBSA) emulsified in complete Freund’s adjuvant (CFA) (10 μl) followed by an intraarticular booster of mBSA (50 μg) in saline (50 μl) administered at 7 and 14 days. The control group received saline (50 μl, ia). After the first intraarticular injection, ten IA animals were supplemented via gavage with Col V (500 μg/300 μl) daily for 30 days (IA/Suppl). The control group received saline (50 μL) and Col V supplement in the same way (Suppl). Col V oral administration in IA/Suppl led to 1) inhibited edema and severe inflammatory cell infiltration, 2) decreased collagen fiber content, 3) decreased collagen type I, 4) inhibited lymphocyte subpopulations and macrophages, 5) inhibited IL-1β, IL-10, IL-17 and TNF-α production and 6) increased expression of caspase-9 in the synovial tissue. In conclusion, Col V supplementation decreased synovial inflammation and the fibrotic response, possibly by increased the apoptosis of inflammatory cells.

## Introduction

The synovium is one of the major tissues affected in most inflammatory joint diseases, including rheumatoid arthritis (RA) [1]. The inflammatory condition of the synovial membrane of the joints is defined as synovitis, which results in *pannus* formation and joint destruction [2]. The treatment for this inflammatory disorder is based mainly on the use of cytotoxic drugs, which affect all proliferating cell types [3]. In this respect, the attempt to decrease the inflammatory process with collagen type V (Col V) supplementation seems to be promising and without troublesome toxicity.

The synovial tissue consists of a modified space, formed by the lining cells of the synovial membrane, which functions as a biological lubricant, as well as a biochemical support through which nutrients and cytokines penetrate and reach the articular surface [4,5]. This tissue rests on a thick frame (~100 nm) of loose subsynovial connective tissue, the extracellular matrix of which consists mainly of collagen type I and a minor proportion of collagen types III, IV, V and VI, chondroitin sulfate, proteoglycans, and fibronectin [6–8]. Synovial inflammation results in a metalloproteinase environment and joint matrix remodeling, exposing neoepitopes of the tissue components. In this context, articular cartilage type II, IX and XI collagens were shown to have arthritic and immunogenic properties [8,9]. Moreover, studies from our group described an intense synovial remodeling process with collagen deposits in rabbits immunized with Col V [10, 11].

Col V, a minor collagen, is intercalated within fibrils of type I collagen [12]. Due to its location, Col V is considered a sequestered antigen not normally exposed to the immune system. However, interstitial remodeling leads to Col V exposure and results in a cryptic antigen becoming a target of the immune response. In recent years, the fact that Col V becomes a neo-autoantigen after inflammatory processes involving tissue remodeling in pathologies, such as obliterans bronchiolitis after lung graft rejection in rats, airway hyper responsiveness, idiopathic pulmonary fibrosis, atherosclerosis and experimental systemic sclerosis, has become important [13–20]. Of note, Col V-induced tolerance suppresses/downregulates the development of these pathologies [13–15, 21, 22].

Because Col V can be exposed in tissue remodeling processes in synovial inflammation, supplementation with this collagen type can be a therapeutic option for the reduction in synovial inflammation and consequent protection of the joint. To test this hypothesis, we employed a well-established arthritis model that utilizes methylated bovine serum albumin (mBSA) and Freund’s Complete Adjuvant (CFA) to induce joint inflammation in rats, with the subsequent development of chronic synovial inflammation, pannus formation and joint destruction [23]. In this study, we supplemented the animals with Col V up to 30 days after joint inflammation induction and verified the profile of the cellular infiltrate, tissue remodeling and pro-inflammatory cytokines [23].

## Materials and methods

### Preparation and characterization of bovine collagen type V

For extraction of bovine collagen, the tissue samples were sliced and washed in EDTA containing proteinase inhibitors (50 mM EDTA, 5 mM phenylmethylsulfonyl fluoride and 0.02 Mm, N-ethylmaleimide) for 48 hours at 4°C. The samples were homogenized with a Teckman polytron and centrifuged at 12,000 rpm for 30 minutes. The residue was treated with pepsin (10,000 dry unit/ml; substrate/enzyme 10:1) (Sigma Chemical Co.) in 0.5 M acetic acid and stirred at 4°C for 16 h. The insoluble residue was pelleted by centrifugation (15,000 rpm for 60 min), and Col V was purified from the supernatant by differential NaCl precipitation [24]. Col V was soluble in 0.7 M NaCl and precipitated in 1.2 M NaCl [24]. The collagen chains were separated by SDS-PAGE (7.5%) under reduction conductions with 2-mercaptoethanol and electrotransferred onto a nitrocellulose membrane (Sigma Chemical Co.) [25]. The membrane was blocked in 5% skim milk in phosphate-buffered saline (PBS) for 1 h at room temperature, and the immunoreaction was performed by incubation with rabbit polyclonal anti-collagen type I (1:600; Rockland), mouse monoclonal anti-human type III collagen (1:100; Rockland) and rabbit polyclonal anti-human collagen type V (1:100) [19]. After incubation with alkaline phosphatase-linked anti-rabbit IgG or anti-mouse IgG (1:1,000; Sigma Chemical Co.), the blot was developed using 5-bromo-4-chloro-3-indolyl-phosphate (BCIP) as the alkaline phosphatase substrate and nitro blue tetrazolium (NBT) as the chromogen.

### Animal preparation and experimental protocol

This study was approved by the Ethics in Committee Research for animal studies of University of São Paulo School of Medicine (São Paulo, Brazil) N° 295/12.

Thirty male Lewis rats with an average weight of 220–240 g and an average age of 3 months were used. Food and water were given ad libitum. The animals were kept in a room with a controlled environment and a circadian night–day rhythm of 12 h. All the animals received human care in compliance with the Guide for the Care and Use of Laboratory Animals published by the US National Institutes of Health [26].

**Fig 1:**
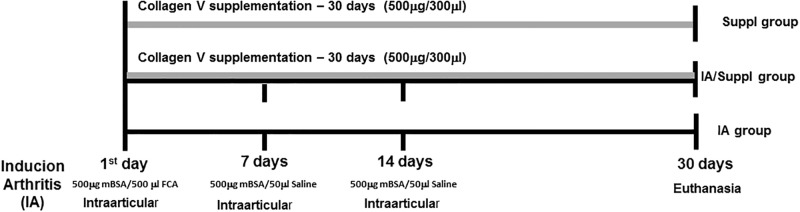
Schematic drawing of mBSA-induced arthritis and Col V oral administration for 30 days.

The arthritis group (IA, n = 20) was induced by intraarticular injection (10 μL) of 500 μg of mBSA (Sigma Chemical Co.) diluted in saline (10 μl) and emulsified in an equal volume of complete Freund’s adjuvant, followed by an intraarticular booster of mBSA (50 μg) in saline (50 μl) administered at 7 and 14 days [27]. After the first intraarticular injection, 10 animals were further randomized to be supplemented via gavage with Col V (500 μg/300 μl) diluted in acetic acid (0.01 N) and administered daily for 30 days (IA/Suppl). The control group (n = 10) received saline (50 μl) using the same protocol and was Col V supplemented in the same way (Suppl) (Fig 1).

### Synovial histology

The synovia were removed from the right knee under strictly sterile conditions, fixed in buffered 10% formalin for 24 hours and embedded in paraffin for light microscopy. Slices (3 μm thick) were cut and underwent Hematoxylin–Eosin (H&E) staining for morphometric analysis of synovial architecture, edema and inflammatory cells (mononucleated and polymorphonucleated cells).

Thick and thin collagen fibers in the synovia were characterized by the Picrosirius-polarization staining method [28]. Sirius red binds selectively to collagen fibers, creating colored products in which the bound dye is proportional to the amount of collagen present [28]. The color intensity is proportional to the collagen concentration, which allows for the measurement of the emitted light during observation using polarized light. The birefringence also reflects the degree of parallel orientation and state of aggregation of the collagenous structures [29].

Subpopulations of lymphocytes [T and B] and macrophages were identified by immunohistochemistry. The sections were deparaffinized, and a 0.3% hydrogen peroxide solution was applied for 4 x 5 minutes to inhibit endogenous peroxidase activity. The antibodies used in the immunohistochemical reactions were CD3 (1:700; Santa Cruz Biotechnology Inc.), CD20 (1:600; Santa Cruz Biotechnology Inc.), CD68 (1:3,200; Santa Cruz Biotechnology Inc.). Tissue sections were pretreated in citrate buffer solution pH 6.0 and heated in a Pascal pressure cooker (125°C for 1 minute) to unmask the epitopes. The sections were incubated with the primary antibody overnight at 4°C. The reaction was revealed using a biotin–streptavidin–peroxidase kit (Vector) according to the manufacturer’s instructions. 3,3 diaminobenzidine (Sigma Chemical, St Louis, MO) was used as a chromogen. The sections were counterstained with Harris hematoxylin (Merck, Darmstadt, HE Germany). For the negative controls, the primary antibody was replaced with PBS.

Collagen type I (Col I) was evaluated by immunofluorescence. The slides were immersed in xylene and dehydrated in decreasing ethanol concentrations. The immunogenic sites were exposed by enzymatic treatment of the synovia samples with bovine pepsin (10,000 UTD; Sigma Chemical Co.; St. Louis, Missouri, USA) at 6 mg/ml acid buffer, pH 2.2, for 30 min at 37°C, followed by incubation in 5% BSA/PBS, pH 7. The slides were incubated overnight at 4°C with rabbit polyclonal anti-collagen type I (Rockland; Immunochemical Inc., Limerick, PA, USA) diluted at 1:50 in PBS. The slides were washed several times in PBS-0.05% Tween 20 and incubated for 90 min with Rabbit Alexa Fluor 488 (Invitrogen) diluted 1:200 in PBS containing 0.006% Evans blue. The slides were again washed several times in PBS-0.05% Tween 20 and mounted with buffered glycerin. The reaction was visualized under an Olympus fluorescence microscope.

Morphometric analysis was done using the *Image-ProPlus 6*.*0* system composed by an Olympus camera (Olympus Co, St Laurent, Quebec, Canada) coupled to an Olympus microscope (Olympus BX51), from which the images were sent to an LG monitor by means of a digitizing system (Oculus TCX, Coreco, Inc., St. Laurent, Quebec, Canada) and downloaded to a computer (Pentium 1330 Mhz). The area fraction of edema, inflammatory cells, lymphocytes T and B and macrophages positive cells in synovial tissue were determined at 400× magnification in 10 random fields by the point-counting technique [30] using a reticulum grid with 100 points distributed orthogonally on the acquired image. The area fraction was calculated as the proportion of the number of points hitting the morphological parameters in synovial tissue and the total grid area was expressed as a percentage. The area occupied by collagen fibers in the synovia was determined at 400× magnification in 10 random fields by the optical density, adjusting the threshold level of measurement up to the density of thick fibers (reddish orange) and down for thin fibers (yellow greenish). The results were calculated by the area of the collagen fibers divided by the total area of the synovia expressed as a percentage.

### ELISA for anti-Col V antibodies

To evaluate the Col V-humoral immunity in the mBSA-induced arthritis model, 96-well microtiter plates (Immunolon II, Thermo Fisher Scientific) were coated with Col V (5 μg/ml), diluted in coating buffer (Tampão Bicarbonato, pH 9.6). Plates were covered and incubated overnight at 4°C. After aspirating the wells and washing with PBS-Tween 20 (0.05% PBS in Tween 20; Sigma-Aldrich), plates were blocked with PBS-1% BSA (Sigma-Aldrich) for 1 h at room temperature. Animal sera samples (100 μl) were added to duplicate wells, incubated for 1 h at room temperature followed by washing with wash buffer. Anti-rat IgG HRP-conjugated Abs (1:1000 dilution; Sigma-Aldrich) was added to each well and aspirated after 60 min of incubation at room temperature. Plates were developed by the addition of TMB Substrate Reagent (BD Pharmingen) to each well (100 μl/well). Reactions were stopped after 30 min of incubation in the dark by the addition of 2 N H_2_SO_4_ to each well, and the absorbance was read at 450 nm.

#### Quantitation of cytokines

Measurements were performed using the Multiplex MAGPIX system (Millipore Corporation, Billerica, MA, USA) using the XPONENT 4.2 software for acquisition and assay Interleukin-1 [IL-1], interleukin-10 [IL-10], interleukin-17 [IL-17] and Tumor Necrosis Factor-Alpha [TNF-α] plasma/serum levels were assayed using Kit Cytokine Milliplex Map Rat Cytokine/Chemokine Magnetic Bead Panel design.

### Statistical analysis

Statistical comparisons were performed using GraphPad Prism 6 (GraphPad Software, Inc., San Diego, CA). The differences among groups were assessed by a one-way ANOVA followed by Tukey’s test. Data are expressed as the mean ± standard error (SEM). A p value less than 0.05 was considered significant.

## Results

### Col V supplementation in induced arthritis decreases the inflammatory process and collagen remodeling

**Fig 2:**
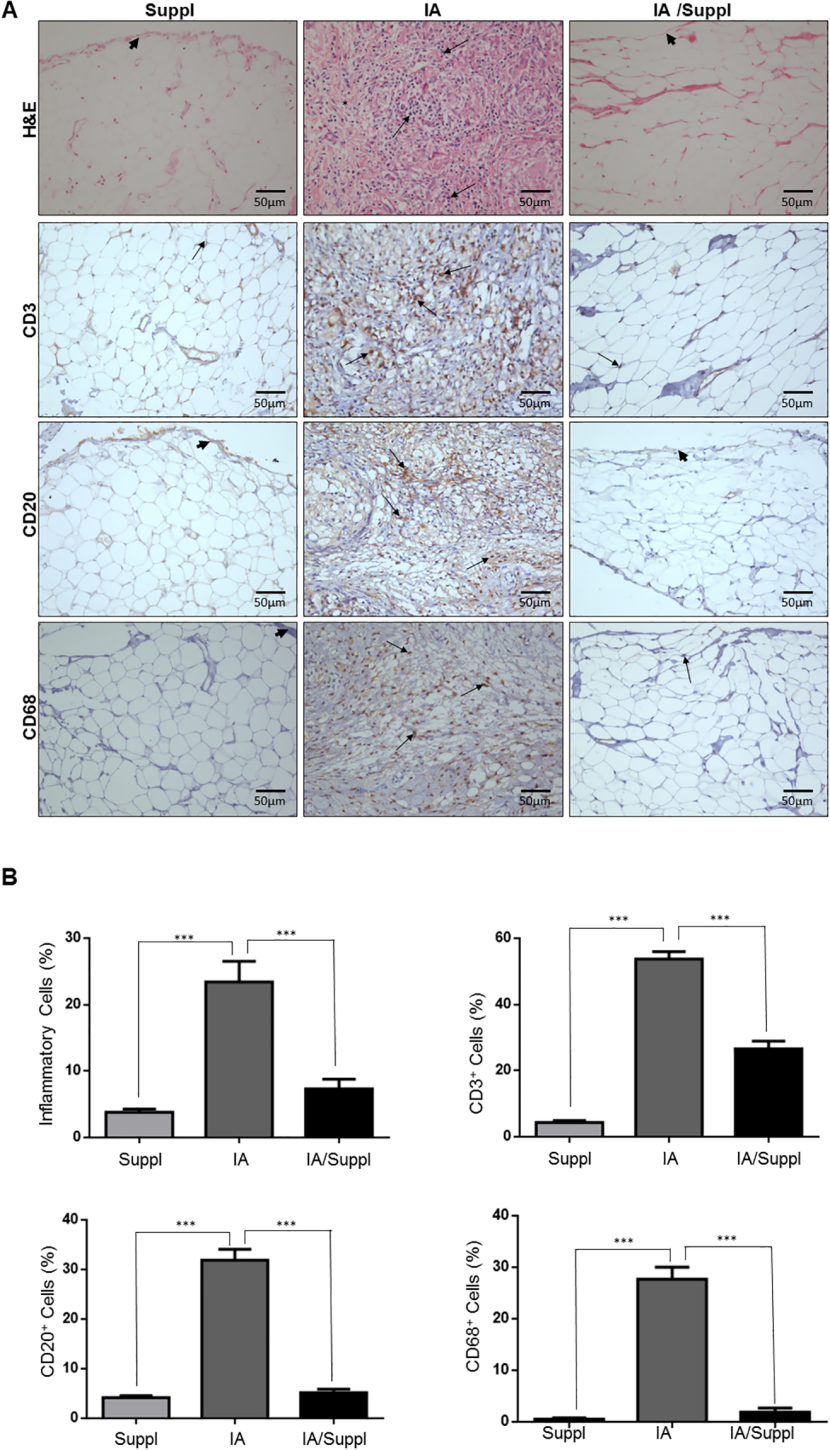
Histological sections of synovial tissue from the IA, IA/Suppl and Suppl groups. Panel (A and B) shows synovial tissue from the Suppl group with vessels and synovial membrane preserved (arrowhead) and scarce inflammatory cells (arrow). The arthritis group (Panel A and B) has prominent edema, synovial membrane inflammation (arrowhead), cellular inflammatory infiltrate and fibrotic thickening (arrow). In contrast, the IA/Suppl (A and B) groups showed significant reductions in edema, inflammatory cell infiltrates (arrows) and fibrotic thickening in addition to synovial membrane preservation (arrowhead). Panels A and B show peroxidase immunostaining of CD3+ (T lymphocytes), CD20+ (B lymphocytes) and CD68+ (macrophages) in the IA, IA/Suppl and Suppl groups. The synovial tissue of the control shows scarce CD3+ (E), CD20+ and CD68+ (A and B) cells. The immuno-expression of CD3+, CD20+ and CD68+ cells (arrows) in the synovial tissue of the IA/Suppl and Suppl groups is significantly decreased in relation to the IA group. Panels (A and B), H&E staining. Original magnification; 400×. Values are the means (±SEM) of 10 animals in each group. All values were completed in a few random, now coincident fields supplemented with daily doses for 30 days. Groups are compared using ANOVA. IA vs Suppl (p<0.01), IA vs IA/Suppl (p<0.01), Suppl vs IA (p<0.01).

**Fig 3:**
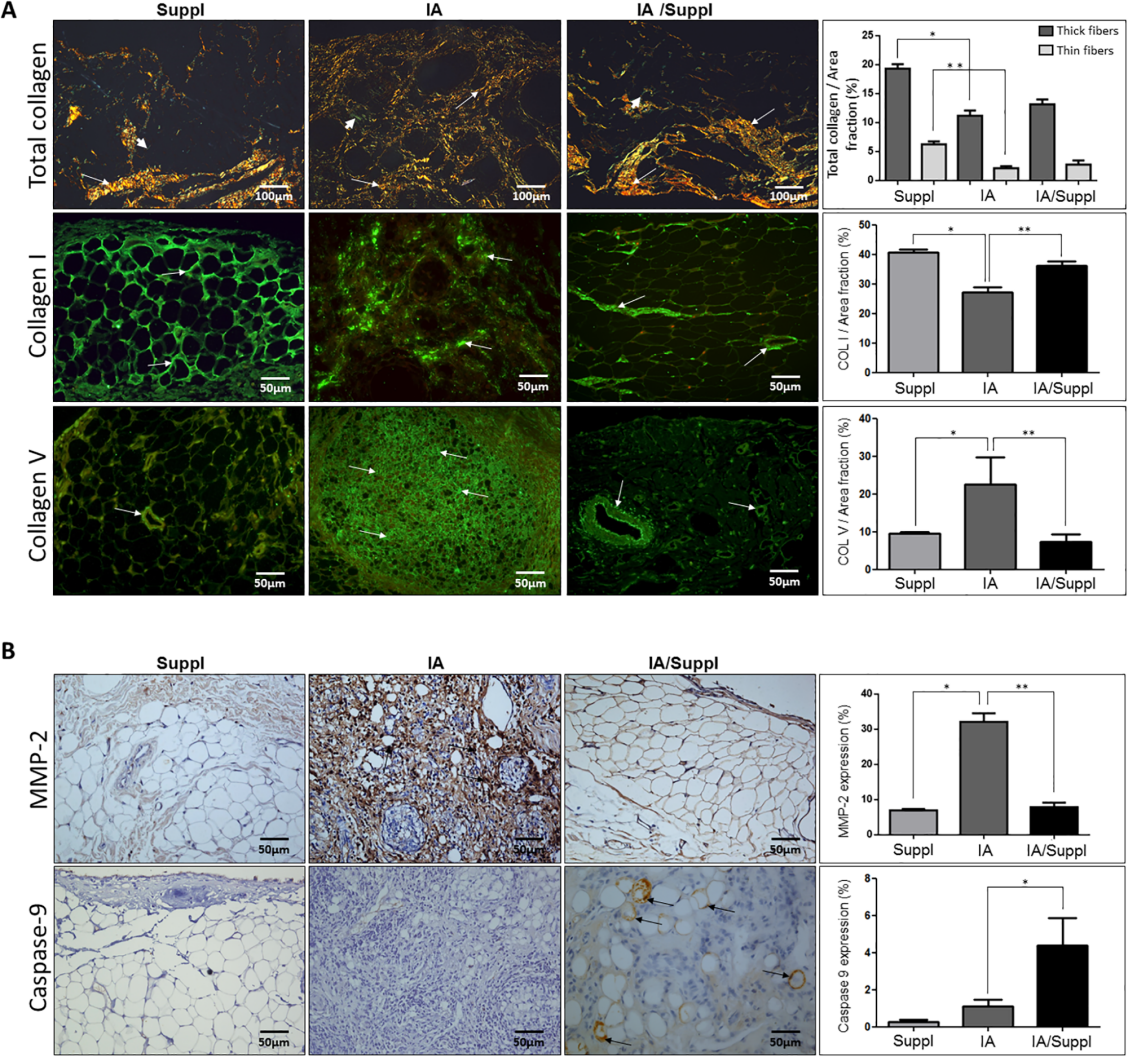
(Panel A) Collagen fibers in the synovial connective tissue, immunofluorescence for collagen I and V fiber expression in the synovia of the IA, IA/Suppl and Suppl groups. The Suppl group shows weak red (thick fibers) and yellow-greenish (thin fibers) (arrow) birefringence, displaying a preserved and normal distribution of collagenous fibers in the synovial membrane (arrowhead) and subsynovial compartment. The IA group shows a predominance of thickened collagen fibers in the synovial membrane (arrowhead) and subsynovial compartment (arrows). The immunofluorescence of Col V fibers showed this collagen in the synovial membrane (arrowhead) and subsynovial compartment (arrows). The Suppl and IA/Suppl groups presented decreased Col V expression compared to the IA group in the synovial adjacent tissue (arrows) and synovial membrane (arrowhead). The immunofluorescence of collagen I fibers showed this collagen in the synovial membrane (arrowhead) and subsynovial compartment (arrows). The Suppl and IA/Suppl groups presented increased collagen I expression compared to the IA group in the synovial adjacent tissue (arrows) and synovial membrane (arrowhead). (Panel B) Immunostaining of metalloproteinase 2, and caspase-9 in the IA, IA/Suppl and Suppl groups. The Suppl and IA/Suppl groups show scarce metalloproteinase 2 expression compared to the IA group in the synovial adjacent tissue (arrows) and synovial membrane (arrowhead). The immuno-expression of caspase-9 cells (arrows) in the synovial tissue of the IA/Suppl is significantly increased in relation to the IA group. Picrosirius staining visualized by polarized light; I-L: Immunofluorescence staining. Original magnification: A-H, 200×; I-L, 400×. Values are the means (±SEM) of 10 animals in each group. All values were completed in a few random, now coincident fields supplemented with daily doses for 30 days. Groups are compared using ANOVA. IA vs Suppl (p<0.01), IA vs IA/Suppl (p<0.01), Suppl vs IA (p<0.01).

Histopathological examination showed that mBSA animals (IA) (Fig 2A) presented prominent edema, inflammatory cell infiltration and collagen fiber degradation compared to other groups. The area occupied by edema and inflammatory cells along the synovial membrane (Fig 2A and 2B) was significantly increased in arthritis animals. In fact, the synovial membrane of arthritis animals presented a significant number of macrophages CD68+, lymphocytes CD20+ and lymphocytes CD3+T compared to other groups. Conversely, a significant increase in Col V and significant decreases in thick collagen fibers and Col I were observed in the synovial membrane of the IA group compared to that of the control group (Fig 3A). Histologically, Col V oral supplementation attenuated inflammation and edema in the synovial membrane in the IA/Suppl group compared to the IA group (Fig 2A and 2B), coinciding with a significant decrease in the number of CD68+ macrophages, CD20+ lymphocytes and CD3+ lymphocytes (Table 1). Interestingly, Col V oral administration significantly decreased the volume of Col V fibers in synovial membrane of the IA/Suppl group compared to the IA group, and the volume was similar to that of the control group (Table 1, Fig 3A). No significant difference was observed in the volume of Col I fibers in the synovial membrane between the control and IA/Suppl animals (Table 1, Fig 3A).

**Table 1:** Quantitative distribution of histological and immunohistochemical parameters in the synovia according to the groups studied.

PARAMETERS	SUPPL	IA	IA/SUPPL
**Inflammatory cells**	3.79 ± 1.59*	23.42 ± 10.26*†	7.32 ± 4.09†
**Edema**	4.15 ± 2.86*	40.82 ± 13.40*†¶	17.42 ± 3.21†¶
**Lymphocytes T (CD 3 +)**	40.34 ± 1.55*	53.74 ± 7.07*†¶	26.59 ± 7.03†¶
**Lymphocytes B (CD 20+)**	4.14 ± 1.40*	31.89 ± 7.10*†	5.19 ± 2.27†
**Macrophages (CD 68+)**	0.53 ± 0.49*	27.67 ± 7.46*†	1.87 ± 2.27†
**Thick collagen**	6.25 ± 1.35*	2.12 ± 1.01*†¶	2.75 ± 2.02†¶
**Thin collagen**	19.32 ± 1.86*	11.19 ± 2.83*†¶	13.16 ± 2.71†
**Collagen type I**	40.70 ± 1.45*	27.10 ± 4.26*†	36.19 ± 3.00†
**Collagen type V**	9.51 ± 0.83	22.47 ± 12.42†	7.33 ± 4.15†
**Metalloproteinase 2 (MMP2)**	6.98 ± 1.24*	32.10 ± 7.36* †	7.86 ± 3.96†
**Caspase-9**	0.25 ± 0.43¶	1.10 ± 1.01	4.40 ± 3.87†
**Interleukin IL1β+**	1.31 ± 1.69*	21.96 ± 12.29*†	4.28 ± 4.75†
**TNF α+**	1.70 ± 0.51*	18.61 ± 9.74*†	1.76 ± 0.39†
**Interleukin IL17+**	0.73 ± 0.85*	27.24 ± 10.65*†	2.19 ±0.50†
**Interleukin IL10+**	1.31 ± 1.69*	27.95 ± 17.11*†	0.13 ± 0.03†

Values are the means (±SEM) of 10 animals in each group. All values were computed in ten random, non-coincident fields per rat. Inflammatory cells, edema, cytokines and caspase 3 are expressed as the number occupied by area/1 mm^2^. Collagen fibers are expressed as the occupied volume/mm^3^. The control group received saline (50 μL). In the Suppl and IA/Suppl groups, rats received 300 μl of Col V (500 μg) diluted in acetic acid (0.01 N) daily (Suppl) and at 30 days (IA/Suppl) via gavage.

*IA vs Suppl (p<0.01),

^†^IA vs IA/Suppl (p<0.01),

^¶^ Suppl vs IA/Suppl (p<0.01) [ANOVA]

### MMP-2 and pro-inflammatory cytokines decrease in induced arthritis after Col V supplementation

**Fig 4:**
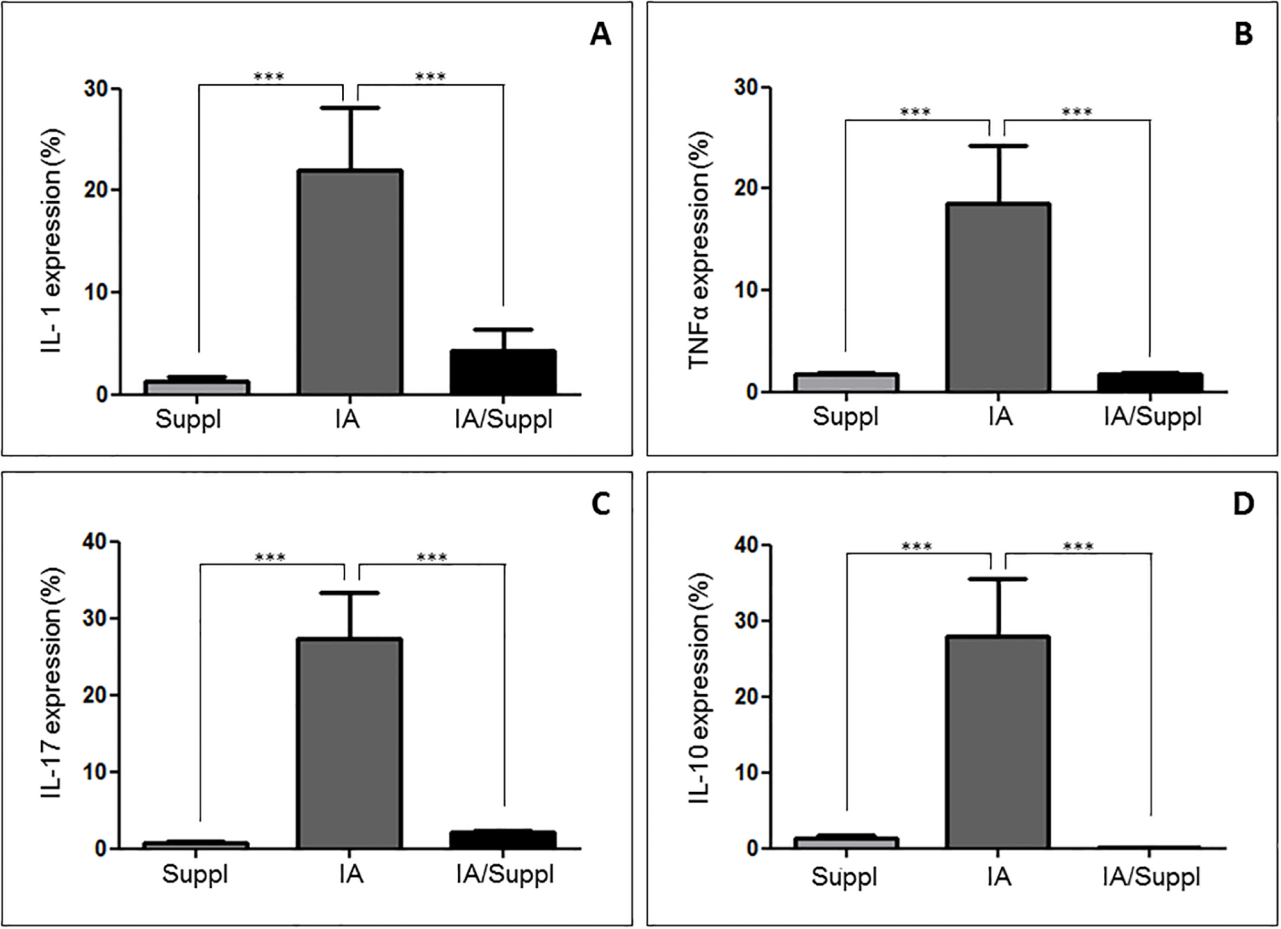
Immuno-expression of IL-1β, IL-10, IL-17 and TNFα in the synovial sera from the IA, IA/Suppl and Suppl groups. Values are the means (±SEM) of 10 animals in each group. All values were completed in a few random, now coincident fields supplemented with daily doses for 30 days. Groups were compared using ANOVA. IA vs Suppl (p<0.01), IA vs IA/Suppl (p<0.01), Suppl vs IA (p<0.01).

**Fig 5:**
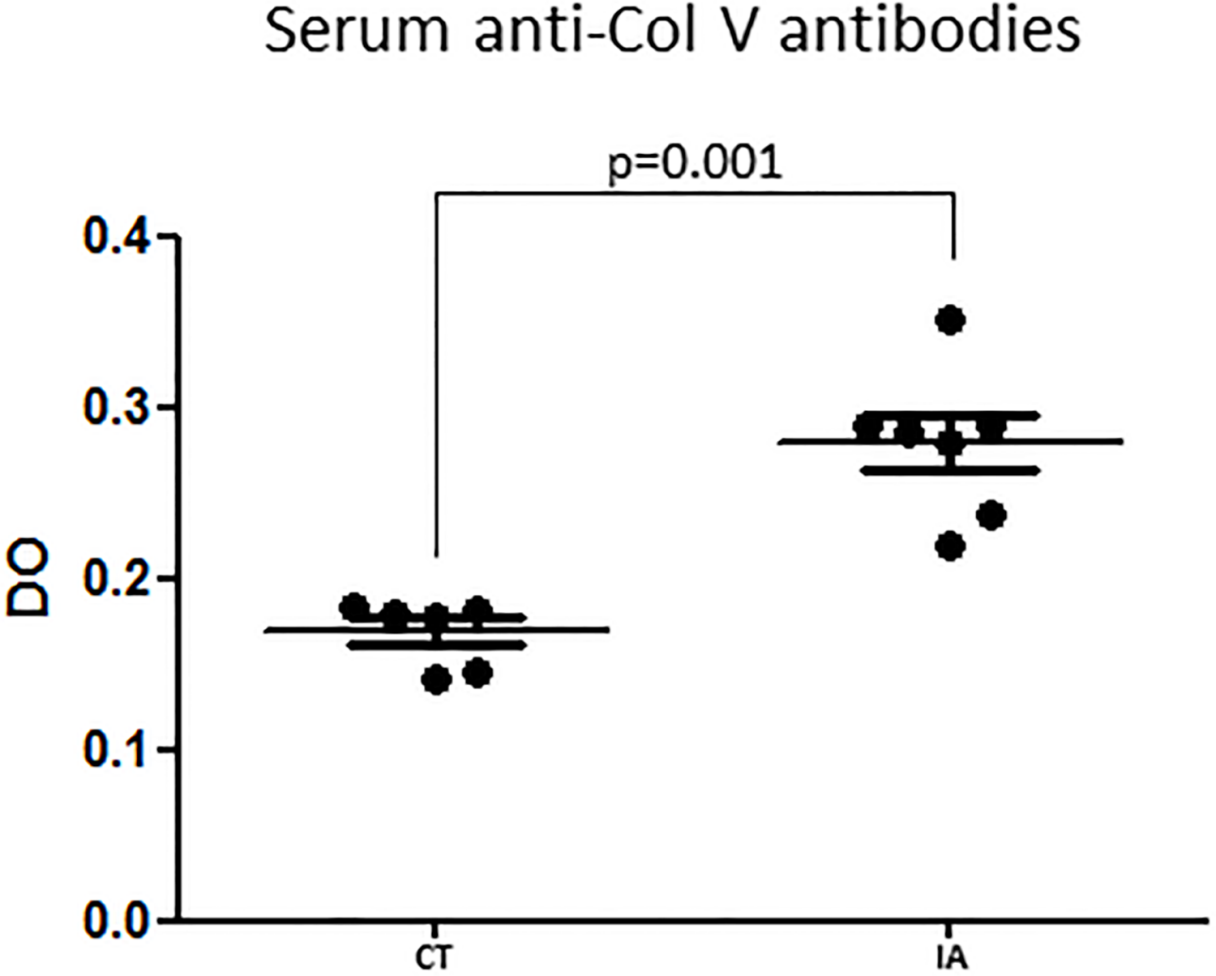
Serum anti-Col V antibodies level in the CT and IA groups 30 days after the first intraarticular injection of saline (CT) and methylated bovine serum albumin antigen (AI). Groups are compared using ANOVA. CT vs IA (p = 0.001).

Fig 3B shows that MMP-2 expression was increased in the synovial membrane of the IA group compared to the other groups. This finding coincided with a significant decrease in collagen thick fibers and type I collagen (Table 1, Fig 3A). In contrast, after Col V supplementation, the IA/Suppl group presented a significant decrease in MMP-2 (Table 1, Fig 3B). The IA/Suppl and Suppl groups presented no significant difference in MMP-2 expression. Furthermore, in addition to the increase in the cellular inflammatory process, the IA group presented a significant increase in IL-1β, IL-17 and TNF-α pro-inflammatory cytokines levels (Fig 4), associated with a significant increase in serum circulating anti-Col V antibodies (Fig 5). On the other hand, these pro-inflammatory cytokines decreased after Col V supplementation (Table 1, Fig 4). Interestingly, IL-10 increased in the IA group and decreased after Col V supplementation in the IA/Suppl group (Table 1, Fig 4).

### Caspase-9 increased after Col V supplementation

Immunohistochemical staining of the synovial membrane showed increased caspase-9 in the vessels of the IA/Suppl group compared to those of the IA group (Table 1, Fig 3B). This finding coincided with a decrease in MMP-2 and pro-inflammatory cytokines in the IA/Suppl group after Col V supplementation (Table 1, Figs 3B and 4).

## Discussion

To our knowledge, this is the first study to evaluate the effects of Col V on synovial inflammation and remodeling in experimental arthritis induced by methylated bovine serum albumin antigen. This current model, which was developed in our laboratory, presented histological changes of the synovial membrane that resembled human synovitis with prominent edema, dense inflammatory cell infiltration, the release of cytokines and collagen fiber degradation by MMP-2. Interestingly, after 30 days with collagen V oral administration, there was a significant attenuation of the inflammatory response in the synovial membrane, with decreases in immune cell recruitment and pro-inflammatory cytokine release, increased cell apoptosis and the restoration of collagen fibers.

In the present study, the arthritis model was induced by intraarticular injection of an emulsion of methylated bovine serum albumin antigen and complete Freund’s adjuvant, which promoted a pronounced recruitment of immune cells and fibrotic activation, in contrast with other arthritis models, such as those induced with Zymosan and Collagen II [31–33]. In the mBSA arthritis model, 30 days after the induction the inflammatory infiltrate in the synovial membrane, the region was rich in CD68+ macrophages, CD3+ lymphocytes and CD20+ lymphocytes, culminating with fibroblast proliferation after 45 days [34,35]. According to the literature, in this model, the first injection of mBSA emulsion in complete Freund’s adjuvant, which contains inactivated *Mycobacterium tuberculosis*, activates the immune system, resulting in an influx of macrophages, T and B lymphocytes, as well as albumin/anti-albumin immune complex, which increases with subsequent mBSA reinforcement [36]. Therefore, in this inflammatory microenvironment, the activated macrophages produce a variety of harmful substances, such as toxic oxygen metabolites, proteases (metalloproteinases) and nitric oxide [37]. Additionally, the albumin/anti-albumin immune complexes activate the complement system, increasing inflammation and tissue injury [35,36]. Because Col V is located inside the heterotypic fibrils (I/III/V), when exposed to the immune system, Col V becomes a potential antigen in the inflammatory process [12,16–18]. In fact, we found higher levels of anti-Col V antibodies in the serum of the mBSA-induced arthritis model. Corroborating this finding is the presence of circulating anti-Col V antibodies in the serum of patients with rheumatoid arthritis [38]. Therefore, it is reasonable to assume that autoimmunity to Col V can actuate in inflammation and remodeling in the current arthritis model.

Col V has been identified in several tissues of adults. This collagen type belongs to the fibrillary collagen family, which is ubiquitous in tissues, localized inside the collagen heterotypic fibrils I/III and considered a sequestered antigen [12]. The main functions of Col V are fibrogenesis control and type I collagen fibril diameter regulation [12]. Furthermore, Col V has immunogenic and antigenic properties with potential to become an autoantigen when exposed to the immune system by metalloproteinase actions or through other agents that induce chronic endothelial injury [16–18,39]. In the past few years, Col V has been considered an autoantigen in pathological processes such as lung transplant rejection in mice and humans, heart transplants, idiopathic pulmonary fibrosis, asthma, atherosclerosis and experimental scleroderma [13–18,39]. On the other hand, Col V oral tolerance decreases murine lung graft rejection by eliciting Treg cells and IL10 [21,22]. However, in an atherosclerosis model, induced tolerance by nasal Col V administration decreases atherosclerotic plaque via increased IL35 expression [15]. In an experimental scleroderma model, Col V nasal tolerance decreased cutaneous and pulmonary remodeling, lung cellular infiltrate and fibrotic cytokines [40,41]. Of note, recently, patients with idiopathic pulmonary fibrosis with circulating anti-Col V antibodies in serum were treated by Col V oral immunotherapy. This therapy was well tolerated and improved the pulmonary function of the patients [13].

Although Col V is one of the constituents of synovial tissue, to date, there are few reports in the scientific literature regarding the importance of Col V in synovitis [10,11,38]. Synovitis is currently defined as chronic inflammation with an increase in activated neutrophils, macrophages, and T lymphocytes, leading to *“pannus”* formation and joint destruction [42]. Similarly, in the present arthritis model, the increase in “*pannus”* formation was associated with dramatic inflammatory cell infiltration in the synovial membrane. Afterward, Col V oral administration in the IA/Suppl group reduced inflammation and MMP-2 in line with the increase in collagen I and decrease in Col V fiber deposition, which were comparable to those of the control group. It is known that the development of fibrosis occurs at the expense of the formation of coarse fibers formed by type I collagen. Our results show that the synovial tissue of the IA group showed a decrease in coarse fibers. The decrease in thick collagen fibers is probably related to enzyme activity since this study determined an MMP-2 increase in the IA group. MMP-2, also known as gelatinase A, is one of the key members of a family metalloproteases that are capable of cleaving gelatins; collagen types I, IV, and V; elastin; and vitronectin and is expressed in CD14+ CD68+ monocytes and macrophages that infiltrate the synovial tissue of patients with rheumatoid arthritis [36]. MMP-2 produced by synovial cells such as monocytes and macrophages may play a role in angiogenesis and the progression of rheumatoid arthritis [42–43]. Our data suggest that the remodeling of collagen is due to the high expression of MMP-2 found in the IA group, probably produced largely by macrophages present in the greater proportion in this group. However, there are still some questions. The greatest expression of Col V, present in the IA group, would be due to increased synthesis of this protein in an attempt to repair tissue or by increasing the expression of immunogenic epitopes exposed by intense tissue remodeling. Future studies are needed to clarify this issue.

Another factor of great importance in our study was the reduction in the expression of pro-inflammatory cytokines, such as IL-1β, TNF and IL-17, after Col V supplementation. This fact presumably is associated with reduced inflammatory cells in the IA/Suppl group, which was submitted to Col V oral administration from the induction of experimental arthritis to thirty days. IL-1β is a pro-inflammatory cytokine that is related to stimulation of the proliferation of synovial cells, resulting in the formation of strong cellular infiltration, "pannus". This cytokine can also be related to the production of chemokines such as macrophage inflammatory protein (MIP-2) and IL-8 [43]. Whereas IL-1β is the main cytokine involved in the destruction of cartilage and bone [44], this finding is of great importance for a possible therapy for arthritis. Presently, TNF is considered the primary determinant of the cytokine response in inflammatory arthritis since it promotes the main determinants of disease pathogenesis: bone and cartilage destruction by the activation of osteoclasts and chondrocytes and *pannus* formation by endothelial cell activation and the expansion of cytokines [44,45]. Whereas TNF and IL-1β share some pro-inflammatory activity in arthritis, the reduction in these cytokines after 30 days of Col V supplementation suggests that this might be an adjunct to therapy for arthritis. Furthermore, IL-17 is no less important in the pathogenesis of arthritis and is produced by Th17 cells (CD3+, CD4+) and found in inflamed synovia [45]. The involvement of IL-17 in the induction of inflammation includes the migration of neutrophils to the joint and increased production of IL-1β, TNF, IL-6, chemokines and MMPs [45]. Although Col V oral administration is also effective for the reduction in IL-17 in our induced arthritis model, the IL-1β and TNF cytokines seem to be a therapeutic target of particular interest in the treatment of arthritis. Furthermore, in our study, IL-10 was inhibited in the IA/Suppl group, possibly related to a lower number of B lymphocytes. It is known that cytokine secretion, such as IL-10, by B lymphocytes is related to immune response amplitude regulation. In arthritis, B cells produce IL-10, which is involved in negative regulation as well as in experimental autoimmune encephalomyelitis, inflammation, and the intestinal T-dependent antigen response [46,47].

It is important to highlight the action of Col V oral administration in the decreasing remodeling of collagen fibers in subsynovial tissue and decreasing inflammatory cells and pro-inflammatory cytokines in the IA/Suppl group. Although our main goal has not been to study the mechanisms involved in the induction of oral tolerance with Col V in our model, we observed a significant increase in caspase-9 in the IA/Suppl group. It is well established that the primary factor that determines which form of peripheral tolerance develops after the oral administration of antigens is the dose of antigen administered. Oral administration of low doses of antigen induces the activation of antigen-specific Treg cells, which produce anti-inflammatory cytokines such as IL10 and TGF-β. On the other hand, the oral administration of large amounts of antigen results in the absence of Th1 and Th2 cell responses by clonal anergy or deletion [48–50]. In fact, we propose that the oral administration of large amounts of Col V (500 μg) daily for thirty days could be associated with increased apoptosis of inflammatory cells in the IA/Suppl group. The link between clonal deletion events following oral administration of large amounts of antigen was demonstrated. Phagocytosis of apoptotic cells by macrophages results in decreased production of pro-inflammatory cytokines.

The immunomodulatory role of Col V in the IA model will be better evaluated in future approaches. In this respect, it is relevant to study the synergistic effect of Col V oral administration with granulocyte macrophage colony stimulating factor (GM-CSF) in stimulating peripheral tolerance in the mBSA arthritis model [51,52]. Recently, a study described a pleiotropic effect of GM-CSF, classically recognized as having a pro-inflammatory effect, in regulating the immune response and maintaining immunological tolerance [52,53]. GM-CSF is capable of suppressing many autoimmune diseases, such as Crohn’s disease, Type-1 diabetes, Myasthenia gravis and experimental autoimmune thyroiditis [53,54].

As previously mentioned, our study has limitations since our results support that oral Col V supplementation in the arthritis-induced mBSA model triggers a process of oral tolerance for Col V. However, this collagen type itself has become an antigen in tissue remodeling as a consequence of the inflammatory process with the induction of circulating anti-Col V antibodies. Further studies are needed to demonstrate cellular immunity to Col V in an arthritis-induced mBSA model and whether Col V supplementation stimulates Treg cells and immune-regulatory cytokines.

We conclude that Col V oral administration has an inhibitory effect against the severity of synovial inflammation and collagen remodeling, suggesting that this supplementation can be a support in arthritis treatment.

## Supporting information

S1 Appendix Raw data set of inflammatory process evaluations, collagen quantification, MMP2, Caspase 9 and cytokines expression. (PDF)

## Abbreviations

BCIP: 5Bromo-4chloro-3indolyl-phosphate
BSA: Bovine Serum Albumin
CD20: Cluster of Differentiation 20
CD3: Cluster of Differentiation 3
CD68: Cluster of Differentiation 68
CFA: Complete Freund’s Adjuvant
CGRP: Calcitonin Gene-Related Peptide
Col I: Collagen type I
Col V: Collagen type V
EDTA: Ethylenediaminetetraacetic acid
H&E: Hematoxylin & Eosin
IL-1: Interleukin-1
IL-10: Interleukin-10
IL-17: Interleukin-17
mBSA: methylated Bovine Serum Albumin
MIP: 2 Macrophage Inflammatory Protein
NBT: Nitro Blue Tetrazolium
PBS: Phosphate-Buffered Saline
RA: Rheumatoid Arthritis
TNF-α: Tumor Necrosis Factor-Alpha
