# Supplementary material for: Collagen V oral administration decreases inflammation and remodeling of synovial membrane in experimental arthritis
Source: PLoS One. 2018 Jul 30;13(7):e0201106. doi: 10.1371/journal.pone.0201106 (PMC6066207; doi:10.1371/journal.pone.0201106)
Supplement: S1 Appendix — (PDF) [file pone.0201106.s001.pdf]

INFLAMMATORY CELL

Suppl	AI	AI/Suppl
7,25	38,50	1,09
5,27	19,11	4,83
3,90	35,51	11,39
4,31	36,47	14,86
2,80	14,86	4,25
3,29	12,27	3,26
2,85	14,44	2,29
3,03	21,86	6,08
1,41	30,76	14,64
3,85	10,98	8,33
	22,81	9,57

EDEMA

Suppl	AI	AI/Suppl
4,60	40,71	20,44
1,81	16,28	16,57
2,22	59,97	17,28
0,95	41,08	17,07
0,80	31,27	14,63
3,95	53,42	17,73
5,20	43,69	13,31
4,71	54,69	24,12
9,13	26,78	15,67
8,22	40,32	16,20

CD-3 Expression

Suppl	AI	AI/Suppl
4,69	49,83	31,63
3,60	51,41	19,08
4,53	41,69	30,69
5,77	58,34	28,48
4,53	54,69	26,08
1,11	55,00	25,00
6,26	62,63	34,45
4,25	45,13	31,59
	54,57	12,29
	64,06	

CD-20 Expression

Suppl	AI	AI/Suppl
6,36	26,16	4,42
5,28	27,49	1,29
5,18	31,38	8,22
5,73	35,93	7,67
3,49	28,09	3,22
3,78	40,31	4,24
2,76	25,65	7,66
2,66	48,30	6,98
3,87	32,49	3,54
2,36	28,74	6,28
	26,28	3,62

CD-68 Expression

Suppl	AI	AI/Suppl
0,44	22,57	2,51
0,63	24,32	1,67
0,90	19,03	0,00
0,00	26,38	0,00
0,00	23,03	0,83
1,23	22,72	2,00
0,00	39,02	0,00
0,00	35,45	2,66
0,00	39,63	7,22
	24,51	

Morphometric evaluation

Suppl/ THICK FIBERS	Suppl/ THIN	AI /THICK FIBERS	AI/ THIN	AI/Suppl THICK FIBERS	AI/Suppl THIN
16,425910	7,702762	8,795013	1,323189	11,534920	3,271857
21,181610	6,679328	12,700050	1,838061	13,586690	1,467191
18,543750	6,889996	8,001597	1,010207	18,799510	5,493134
19,969780	6,884322	12,530320	2,643584	15,578540	1,684656
18,529310	5,684024	11,281330	3,140776	12,970950	5,888759
21,288600	3,504873	14,152790	3,891410	8,702916	0,5842276
	6,455498	14,612710	1,900464	14,396210	6,400536
		8,260099	1,273918	12,284950	1,836897
		14,155570		12,396130	1,570980
		7,393711		11,352580	5,739239

COLLAGEN I Quantification

Suppl	AI	AI/Suppl
53,652580	24,50433	32,9897
60,649360	27,41000	24,5000
39,671500	21,79453	28,3800
78,027600	28,80000	36,1100
41,727940	32,98000	35,4400
		40,2200

COLLAGEN V Quantification

Suppl	AI	AI/Suppl
9,508237	36,712220	9,282611
10,130370	13,907590	10,966340
8,347500	16,786120	1,441582
10,087950	17,613000	7,630000
10,055000	14,980010	9,987002

MMP-2 Expression

Suppl	AI	AI/Suppl
7,87	26,56	9,03
6,88	27,00	3,79
4,82	44,30	3,96
7,79	40,85	4,94
8,61	27,82	5,19
5,53	37,49	13,91
8,14	22,01	5,73
7,54	32,35	12,76
6,89	30,56	11,30
5,35		

CASPASE-9 expression

Suppl	AI	AI/Suppl
0,00	0,83	2,08
0,00	1,26	12,06
1,00	0,00	1,87
0,00	0,00	3,00
0,00	2,01	7,09
0,00	2,87	3,31
1,05	1,22	1,39
0,00	1,80	3,01
0,00	0,00	
0,45		

IL-1B – serum quantification (Pg/mL)

Suppl	AI	AI/Suppl
0,54	32,67	5,26
0,44	27,16	3,31
1,74	31,68	0,00
1,18	16,09	1,05
0,65	19,63	3,79
0,59	27,10	3,71
1,85	29,04	2,28
1,02	43,58	1,43
0,78	39,61	1,00
	20,61	1,51
	19,31	1,53

IL-10 – serum quantification (Pg/mL)

Suppl	AI	AI/Suppl
0,90	67,49	6,7700
1,36	46,52	7,4500
3,37	56,30	0,8300
0,00	34,26	2,2727
2,09	49,26	1,0500
0,87	61,40	0,0000
0,00	69,49	0,8700
0,00	43,46	2,8600
0,00	40,99	1,1400
4,18	38,26	0,8300
2,48	32,41	2,6700

TNF Expression

Suppl	AI	AI/Suppl
1,35	14,02	2,26
1,47	29,80	1,99
1,11	12,00	1,22
1,49	13,50	1,61
1,55	15,50	1,75
1,47	14,64	3,25
1,83	22,96	1,35
1,75	14,90	1,61
1,35	13,36	1,99

IL-17 Expression

Suppl	AI	AI/Suppl
0,35	39,54	2,26
0,39	21,09	3,04
0,41	21,12	1,82
3,41	26,00	1,82
0,89	25,60	2,02
0,80	22,04	0,52
0,39	31,08	1,15
0,33	33,06	0,45
0,52	23,07	0,18
